# Early Peritoneal CC Chemokine Production Correlates with Divergent Inflammatory Phenotypes and Susceptibility to Experimental Arthritis in Mice

**DOI:** 10.1155/2019/2641098

**Published:** 2019-02-26

**Authors:** Cristiano Rossato, Layra Lucy Albuquerque, Iana Suly Santos Katz, Andrea Borrego, Wafa Hanna Koury Cabrera, Mônica Spadafora-Ferreira, Orlando Garcia Ribeiro, Nancy Starobinas, Olga Martinez Ibañez, Marcelo De Franco, José Ricardo Jensen

**Affiliations:** ^1^Immunogenetics Laboratory, Butantan Institute, Avenida Vital Brasil, 1500, São Paulo 05503-900, Brazil; ^2^Department of Immunology, Institute of Biomedical Sciences, Universidade de São Paulo, São Paulo 05508-900, Brazil; ^3^União Educacional do Norte, Rio Branco 69915-901, Brazil; ^4^Diagnostics Section, Pasteur Institute, Avenida Paulista, 393, São Paulo 01311-000, Brazil

## Abstract

The inflammatory and autoimmune events preceding clinical symptoms in rheumatoid arthritis (RA) and other autoimmune diseases are difficult to study in human patients. Therefore, animal models that share immunologic and clinical features with human RA, such as pristane-induced arthritis (PIA), are valuable tools for assessing the primordial events related to arthritis susceptibility. PIA-resistant HIII and susceptible LIII mice were injected i.p. with pristane, and peritoneal lavage fluid was harvested in the early (7 days) and late (35 days) preclinical phases of PIA. Chemokine and cytokine levels were measured in lavage supernatant with ELISA, peritoneal inflammatory leukocytes were immunophenotyped by flow cytometry, and gene expression was determined by qRT-PCR. Leukocyte recruitment was quantitatively and qualitatively divergent in the peritoneum of HIII and LIII mice, with an early increase of CC chemokines (CCL2/CCL3/CCL5/CCL12/CCL22) in the susceptible LIII strain. Also, cytokines such as IL-12p40, IL-23, and IL-18 were elevated in LIII mice while IL-6 was increased in HIII animals. The results show that an early peritoneal CC chemokine response is an important feature of arthritis susceptibility and defines potential biomarkers in this model.

## 1. Introduction

Understanding the immunological basis of complex autoimmune diseases such as rheumatoid arthritis is complicated by the fact that patients are almost invariably symptomatic at the time of enrollment in clinical studies. Despite the existence of serological markers such as anticitrullinated peptide antibodies (ACPA), which might be detectable before disease onset (reviewed in [1]), their main value resides in predicting the severity of established disease [2]. As a result, little is known about the early triggers of RA initiation and development.

Therefore, animal models that allow for assessment of the primordial inflammatory and immune events associated with arthritis susceptibility are highly valuable. Pristane-induced arthritis (PIA) [3] is a chronic autoimmune inflammatory disease that shares many immunological and pathological features with human RA. The histology is similar, with thickening of the synovial membrane, pannus, and bone/cartilage destruction in the late stages of the disease [4]. PIA is also characterized by hypergammaglobulinemia, positivity for rheumatoid factor (RF), and antibody/T cell reactivity against a wide range of both joint and ubiquitous antigens [5].

The peritoneal cavity (PerC)—site of pristane injection in mouse PIA—is composed of a complex array of leukocyte populations [6]; their early response to pristane stimulation might diverge in resistant and susceptible mouse strains, resulting in different outcomes of subsequent autoimmune reactions. Pristane injection in the PerC induces chronic IL-6 production by peritoneal cells [7]; however, the role of this cytokine in PIA is not clear. Increased IL-6 is observed in rheumatoid arthritis and murine PIA, but also in mice protected from the disease by prior gamma irradiation [8]. Pristane also induces a lupus-like syndrome characterized by type I interferon production by peritoneal-infiltrating immature monocytes [9].

Early studies on PIA demonstrated that increased numbers of CD4+ T cells are recruited to the PerC of the susceptible CBA/Ig^b^ mice 20-80 days postpristane injection and susceptibility was restored to irradiated mice only when spleen T cells—more specifically CD4+ lymphocytes—were transferred during the first 3 weeks after pristane injection. This suggests that the early preclinical phase is crucial to disease development [10]. The intestinal microbiota is important in the PIA model, as susceptible mice housed under SPF conditions are refractory to arthritis development, regaining susceptibility after transfer to the conventional environment [11].

Several cytokines and chemokines had their roles established in clinical RA, as well as in PIA and other murine arthritis models. However, their function in the early inflammatory and immune events leading to arthritis IS less well understood, because gene knockout affects their function both before and after the disease.

Mouse strains phenotypically selected for extremely high (HIII) or low (LIII) antibody production against complex antigens [12] or acute inflammation [13] have been successfully used for mapping genetic loci regulating their respective selection phenotypes [14, 15]. These strains also diverge in other traits, such as cancer [16] and PIA [17, 18]. Susceptibility of HIII and LIII mice to PIA is extremely divergent (HIII mice are resistant, while 100% of LIII animals develop severe arthritis within 45-120 days postpristane injection [17]), allowing the study of inflammatory events to take place in the PerC during the preclinical phase. In this regard, previous experiments showed that splenocytes from PIA-susceptible LIII mice produce more IL-12p40 and IL-1*β* than HIII early (4-15 days) after pristane injection [17]. This is possibly the result of a divergent peritoneal inflammatory response to pristane in these mice.

Therefore, we studied the early peritoneal inflammatory events induced by pristane in HIII and LIII mice, aiming at mediators potentially associated with the extreme divergence of these strains in PIA susceptibility. We show that the ability to mount an early chemokine-driven recruitment of inflammatory cells to the PerC correlates with divergent peritoneal cytokine production profiles and severe arthritis in LIII mice.

## 2. Materials and Methods

### 2.1. Animals

Male and female inbred HIII and LIII mice from selection III [14] were used. Mice were 2-4 months old at the time of pristane injection and were bred under conventional conditions at the animal facility of the Immunogenetics Laboratory, Butantan Institute. All procedures were approved by the Institutional Animal Care and Use Committee of the Butantan Institute (CEUAIB protocol nos. 655/09 and 1110/13), and all animals received humane care, according to the criteria outlined in the “Guide for the Care and Use of Laboratory Animals” published by the National Academy of Sciences and the National Institutes of Health.

### 2.2. Pristane Injection

Mice (*N* = 4 − 6 per group) were injected i.p. with 0.5 mL pristane (TMPD) (2,6,10,14-tetramethyl pentadecane, Sigma Chemical Company, Saint Louis, MO). For clinical arthritis induction, two doses were given with a 60-day interval and animals were observed for 120 days after the first injection, while a single dose was given for assessment of the preclinical phase. Control animals were injected with saline.

### 2.3. Peritoneal Lavage

After euthanasia in a CO_2_ chamber, the peritoneal cavity of each mouse was washed with 3 mL unsupplemented RPMI1640 medium. The lavage was centrifuged at 400 × *g* for 5 min/4°C, and the pristane layer was removed by aspiration. The supernatant was aliquoted and stored at -80°C until being used. An aliquot of the cells was counted in Malassez hemocytometric chambers and another was cytospun onto glass slides with a Cytospin™ 4 Cytocentrifuge (Thermo Fisher, Cheshire, UK) and Giemsa-stained for differential counts. The remaining cells were either preserved in RNAlater solution for RNA extraction or resuspended in RPMI+2%FBS for flow cytometry.

### 2.4. Real-Time PCR

Total RNA was extracted from peritoneal cells with the Illustra™ RNAspin Mini Kit (GE Healthcare Lifesciences, Buckinghamshire, UK). Quantification and purity of the samples were measured in a NanoVue spectrophotometer (GE Healthcare Lifesciences), and integrity was determined in the Bioanalyzer 2100 instrument with the RNA 6000 Nano Kit (Agilent, Waldbronn, Germany). Samples were used if their RIN (RNA Integrity Number) was at least 7.0. RNA (250 ng) was reverse transcribed with GoScript Reverse Transcriptase (Promega, Madison, USA) and oligo dT_18_ primers. qRT-PCR reactions were carried out in a StepOnePlus real-time detector using SYBR FAST mastermix (Life Technologies, Foster City, USA) in a final volume of 10 *μ*L. The expression levels of several reference genes (*Rps29*, *Tbp*, *Gapdh*, *Ppia*, and *B2m*) were analyzed for stability with the geNorm VBA applet [18], and the combination of *Ppia* and *B2m* was determined as the most stable. Primer pair sequences are described in Table 1 and were designed with Primer-BLAST (https://www.ncbi.nlm.nih.gov/tools/primer-blast/), with the exception of *Mx1*, which was obtained from PrimerBank (https://pga.mgh.harvard.edu/primerbank/; PrimerBank ID 6996929c1).

Normalized (2^−ΔCt^) gene expression [19] was calculated with DataAssist Software (Thermo Fisher Scientific, Foster City, USA) using the mean of *Ppia* and *B2m* Ct values as reference.

### 2.5. ELISA

Cytokine and chemokine levels were quantified in peritoneal lavage fluid with either OptEIA ELISA sets (BD Biosciences Pharmingen, San Diego, USA), Ready SET-Go! ELISA kits (Affymetrix eBioscience, San Diego, USA) or DuoSets (R&D Systems, Minneapolis, USA) as per manufacturers' instructions. Plates were read in a *μ*Quant Spectrophotometer (BioTek Instruments, Winooski, USA), and the standard curves were determined by 4-parameter logistic equations.

### 2.6. Flow Cytometry

Freshly harvested PerC cells were incubated with ACK red cell lysis buffer, counted in the presence of Trypan Blue, and 5X10^5^ viable cells were incubated with marker-specific antibodies, purchased from BD Biosciences: anti-CD11b (clone M1/70), anti-CD11c (clone HL3), anti-CD3e (clone 145-2C11), anti-CD23 (clone B3B4), anti-CD19 (clone 1D3), and anti-CD5 (clone 53-7.3). Nonconjugated monoclonal antibodies were used as isotype controls. Acquisition was carried out in a FACSCanto™ II cytometer (BD Biosciences), and a minimum of 10000 live events (excluding propidium iodide-positive cells) were analyzed with FlowJo software (FlowJo, Ashland, USA).

### 2.7. Statistical Analyses

Unless otherwise stated, group differences were compared with two-way ANOVA, followed by Bonferroni posttests in Prism v.5.0 (GraphPad Software, La Jolla, USA).

## 3. Results

### 3.1. Early Pristane-Induced Peritoneal Inflammation Is Distinct in PIA-Susceptible and Resistant Mice

**Figure 1:**
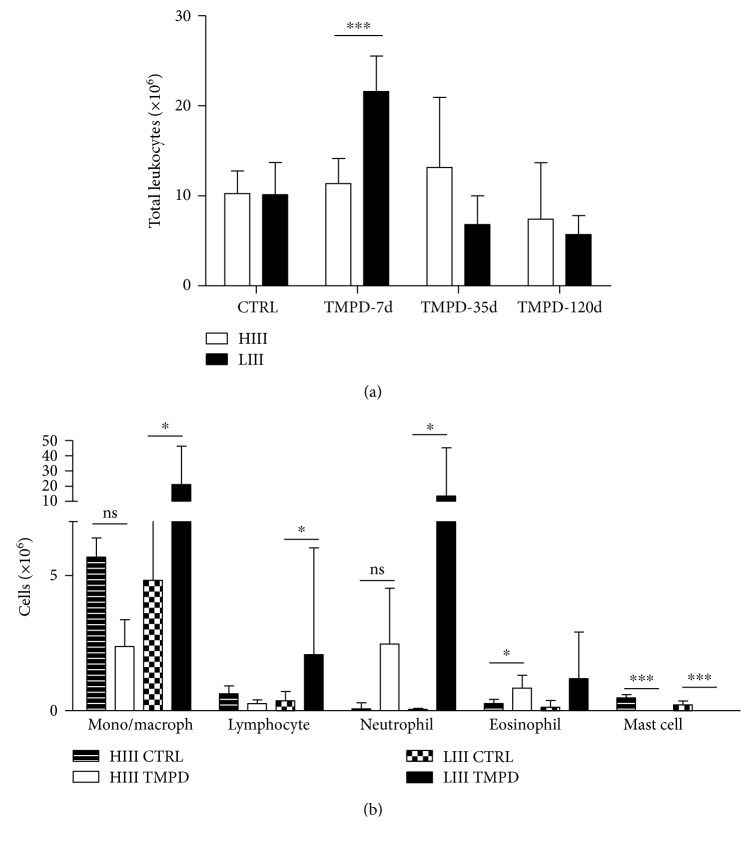
(a) Total leukocyte numbers in the peritoneal cavity of pristane- (TMPD-) injected HIII and LIII mice in the preclinical (7 and 35 days) and clinical (120 days) phases of PIA. (b) Peritoneal leukocyte populations in the early (7 day) preclinical phase of PIA. Bars represent mean ± 95% confidence interval (*N* = 4-6 animals/group; two-way ANOVA followed by Bonferroni posttests). ^∗^ *p* < 0.05, ^∗∗^ *p* < 0.01, and ^∗∗∗^ *p* < 0.001.

The PerC of HIII and LIII control mice is similar in total numbers (Figure 1(a)) and composed by macrophages and lymphocytes, with lower numbers of mast cells and nearly absent neutrophils (Figure 1(b)). Pristane injection induced an early and dramatic shift in the proportions of cells, with striking differences between HIII and LIII mice. The inflammatory infiltrate peaked 7 days postpristane injection in LIII mice, while total cell numbers were similar in pristane-injected and control HIII animals. Mast cells were not detected in the peritoneal lavage of HIII- or LIII-injected mice. In LIII mice, all other cell populations increased significantly, while in HIII mice, despite numerical variation, differences were not significant (Figure 1(b)).

### 3.2. Peritoneal Cell Populations

**Figure 2:**
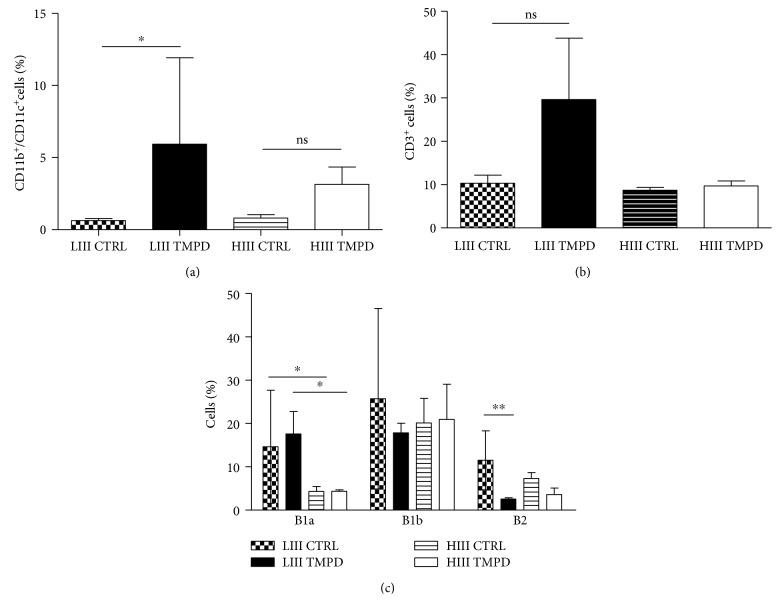
Changes in peritoneal cell subpopulations 7 days postpristane injection in HIII and LIII mice. (a) CD11b^+^/CD11c^+^ DCs, (b) CD3+ T cells, and (c) B1 and B2 Lymphocytes. Data represent two independent experiments (*N* = 4), and bars represent mean ± 95% confidence interval (Kruskal-Wallis test, ^∗^ *p* < 0.05, ^∗∗^ *p* < 0.01, and ^∗∗∗^ *p* < 0.001).

Cytospin preparations showed that alterations in the peritoneal cell populations occurred early after pristane injection. Then, we further investigated whether specific cell subpopulations were enriched or depleted at the 7-day time point, using flow cytometry. Proportions of CD11b^+^/CD11c^+^ dendritic cells (Figure 2(a)) increased only in LIII mice postpristane injection, and while the numbers of CD3^+^ T cells (Figure 2(b)) also increased in this strain, the difference was not significant. The proportions of B2 (CD19^+^/CD23^+^/CD5^−^) and B1b (CD19^+^/CD23^−^/CD5^−^) cells were similar to those in control mice, while B1a (CD19^+^/CD23^−^/CD5^+^) cell percentages were higher in LIII than HIII. Pristane injection reduced the proportion of B2 cells in LIII mice only, while B1a and B1b cell numbers were unaltered (Figure 2(c)).

### 3.3. The Peritoneal CC Chemokine Profile Is Divergent in HIII and LIII Mice during the Preclinical Phase of PIA

**Figure 3:**
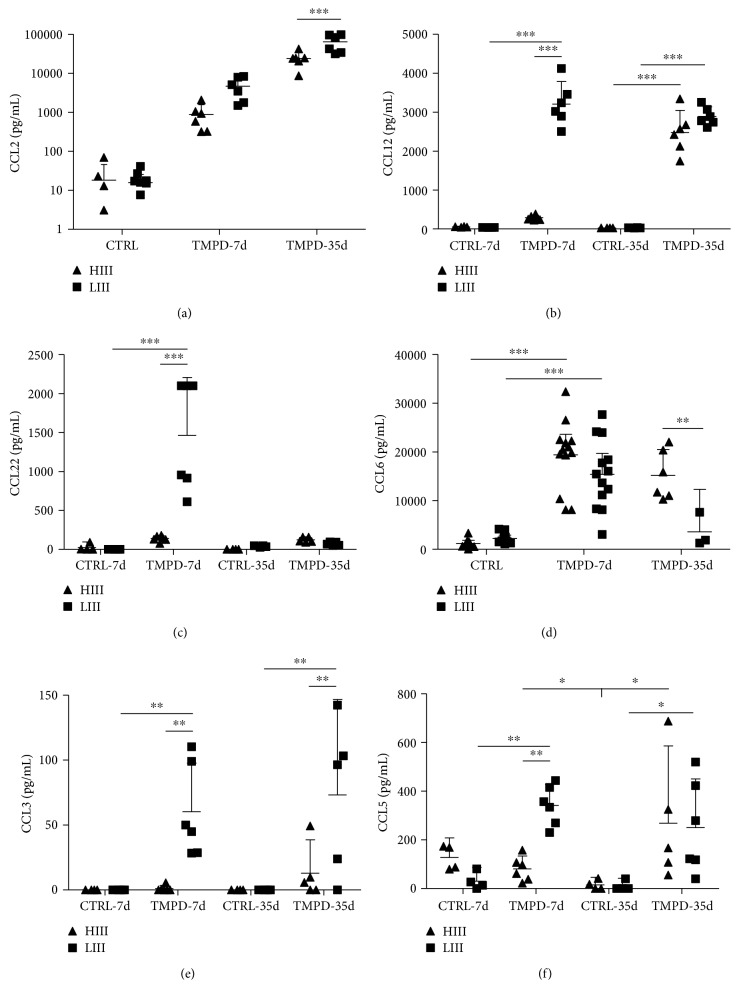
Peritoneal chemokine levels in the preclinical phase of PIA. Chemokine levels were determined in the peritoneal lavage fluid at 7 and 35 days post i.p. pristane injection in HIII and LIII mice. Data are from groups of 4-6 mice (for CCL6, two experiments were combined). Two-way ANOVA with Bonferroni posttests. ^∗^ − *p* < 0.05, ^∗∗^ − *p* < 0.01, and ^∗∗∗^ − *p* < 0.001.

Differences in the PerC mononuclear cell infiltrate of HIII and LIII mice suggested that chemokines might be involved. Then, we investigated peritoneal CC chemokine levels of HIII and LIII mice in the early (7 days postpristane injection) and late (35 days) preclinical phases of PIA. In the early period, levels of all chemokines were significantly elevated in injected LIII mice. On the other hand, only CCL2 and CCL6 levels increased in HIII mice Figures 3(a) and 3(d)), while the levels of the other chemokines were similar to those of the controls.

At 35 days, CCL2 levels rose sharply in both strains, being higher in LIII than in HIII mice (Figure 3(a)). In pristane-treated LIII mice, CCL3, CCL5, and CCL12 levels remained elevated and CCL22 decreased to control levels. In HIII mice, CCL5 and CCL12 levels were similar to those of LIII mice, while CCL3 and CCL22 did not increase above control levels. Elevated CCL6 levels were only observed in HIII mice at this time point (Figure 3(d)).

We also measured the peritoneal levels of the PMN-specific chemokine CXCL1. Pristane induced a significant increase in CXCL1 levels, with similar intensity in HIII and LIII mice (Suppl.  Figure 1a).

### 3.4. Chemokine Receptor Gene Expression in Peritoneal Leukocytes

**Figure 4:**
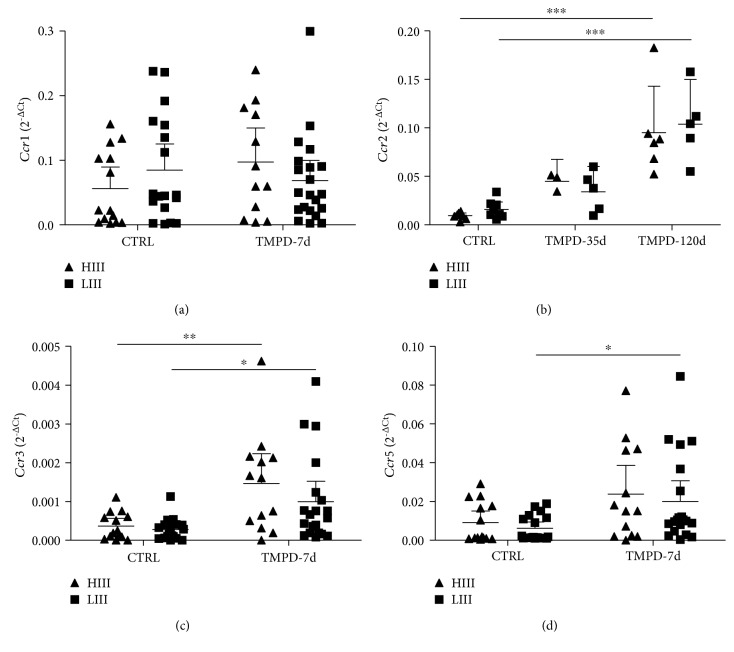
Chemokine receptor gene expression in leukocytes infiltrating the peritoneal cavity of pristane-injected HIII and LIII mice. Data points are the normalized (2^−ΔCt^) expression of each sample. Bars represent mean ± 95% confidence intervals of 2-3 experiments with *N* = 4-6 mice/group (two-way ANOVA with Bonferroni posttests. ^∗^ − *p* < 0.05, ^∗∗^ − *p* < 0.01, and ^∗∗∗^ − *p* < 0.001).

We measured the gene expression of chemokine receptors bound by the chemokines induced by pristane in leukocytes recruited to the PerC of HIII and LIII mice. *Ccr1* expression was similar in injected and control animals (Figure 4(a)), and *Ccr4* transcripts were undetected in any strain irrespective of treatment (results not shown). *Ccr2* and *Ccr3* expression increased in HIII and LIII pristane-injected animals to a similar extent, while *Ccr5* expression increased only in LIII mice 7 days postpristane injection (Figures 4(b) and 4(c)).

We also measured the expression of *Cxcr2*, receptor for the granulocyte-targeting chemokine CXCL1. The expression of this gene increased with similar intensity in both HIII and LIII mice 7 days postpristane injection (Suppl.  Figure 1b).

### 3.5. Peritoneal Cytokine Production in HIII and LIII Mice

**Figure 5:**
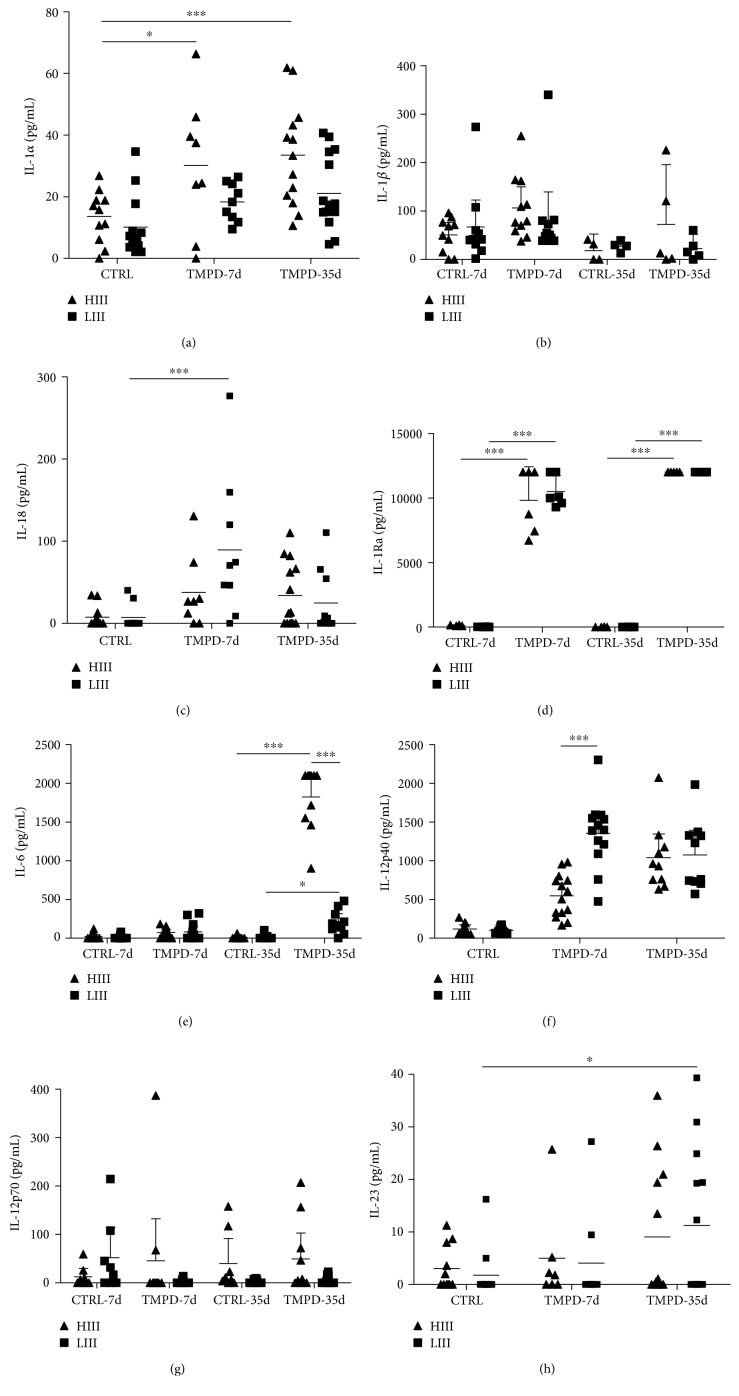
Peritoneal cytokines in the preclinical phase of PIA. Cytokine levels were determined in the peritoneal lavage fluid at 7 and 35 days postpristane injection in HIII and LIII mice. Bars represent mean ± 95% confidence intervals of 1-2 experiments with *N* = 4-6 mice/group (two-way ANOVA with Bonferroni posttests. ^∗^ − *p* < 0.05, ^∗∗^ − *p* < 0.01, and ^∗∗∗^ − *p* < 0.001).

Peritoneal levels of IL-1 family cytokines (IL-1*α*, IL-1*β*, IL-18, and IL-1Ra) diverged in pristane-injected mice during the preclinical phase of PIA. IL-1*β* levels were not altered 7 and 35 days after pristane injection. IL-1*α* levels were higher in HIII animals, while IL-18 production increased in LIII mice only, at 7 days (Figures 5(a) and 5(c)). IL-1Ra levels increased in all injected mice at 7 and 35 days (Figure 5(d)). In pristane-injected mice, IL-6 levels were similar to controls at 7 days; however, at 35 days, levels in HIII mice were significantly higher than those in LIII animals (Figure 5(e)). On the other hand, IL-12p40 levels increased only in PIA-susceptible LIII mice, at 7 days (Figure 5(f)). IL-12p40 is a subunit of both IL-23 and IL-12p70; therefore, both cytokine levels were also measured. IL-12p70 levels were unaffected by pristane treatment, while IL-23 levels were elevated at 35 days in LIII mice (Figures 5(g) and 5(h)).

TNF-*α* levels were higher in HIII than LIII mice at 35 days (Suppl.  Figure 2c). However, this difference may be attributed to two samples with very high values. IL-4, IL-17, and IFN-***γ*** levels were unaltered by pristane treatment (Suppl.  Figure 2a-b-d).

## 4. Discussion

PIA is a late onset arthritis model. However, early pristane-induced production of inflammatory mediators by resident and infiltrating peritoneal cells likely shapes the subsequent autoimmune response by recruiting and activating inflammatory cells. These cells would in turn migrate to secondary lymphoid organs and activate autoreactive helper T cells. On the other hand, resistance would be associated with a quantitatively or qualitatively divergent response to pristane. We characterized the early peritoneal inflammatory response in mouse strains with extreme divergence in PIA susceptibility to investigate this hypothesis.

Our results showed that, during the early preclinical phase of PIA, the levels of several CC chemokines increased in the PerC of LIII mice only. This differential response correlated with distinct inflammatory infiltrates, altered expression of chemokine receptor genes, and divergent production of inflammatory cytokines by infiltrating leukocytes. The CC chemokines target predominantly nongranulocyte cells [20], which might explain the intense monocyte/macrophage, DC, and lymphocyte infiltrate in LIII animals in this early phase. On the other hand, the neutrophil recruitment observed in HIII mice is likely the result of a predominant CXCL1 response.

CCL2 recruits monocytes, macrophages, DCs, eosinophils, and various T cell subsets via CCR2 or CCR4. The CCL2 levels increased similarly in both HIII and LIII mice during the early preclinical phase of PIA, as well as *Ccr2* expression by infiltrating cells, while *Ccr4* mRNA levels were unaltered. CCL2 and CCR2 are extensively studied in rheumatoid arthritis [21, 22]. Increased joint CCL2 levels are also detected in murine arthritis models [23–25]. In collagen-induced arthritis (CIA), CCR2 and CCL2 are elevated in joints but CCR2 neutralization exacerbates disease in this model [26].

CCL12, a mouse-specific chemokine, also binds specifically to CCR2, and little is known about the role of this chemokine in clinical RA and murine experimental arthritis. CCL12 is produced by RANKL-induced osteoclasts [27] and expressed in the synovium of DA rats with PIA [28]. Levels of this chemokine increased in the 7-day peritoneal lavage fluid of LIII mice only, a finding not previously reported to our knowledge.

CCL3 targets diverse cell types, with the exception of neutrophils, by binding CCR1/4/5. Anti-CCL3 treatment did not reduce CIA severity in mice but decreased bone resorption, possibly by impairing osteoclast differentiation [29]. Therefore, CCL3 seems to play a role in the later phases of this model. Early production of CCL3, as well as CCL2 and cytokines such as IL-1*β*, IL-6, IL-12, and IL-1Ra, was observed in an acute antigen-induced peritonitis model [30], showing that peritoneal-resident cells are an important source of these mediators. The increased CCL3 levels and higher *Ccr5* expression in LIII mice suggest that this chemokine also plays a role in early arthritis development, at least in the PIA model.

CCL22, secreted by monocytes, macrophages, and dendritic cells, has been recently described as one abundant chemokine in the synovium of clinical RA, as opposed to non-RA synovium [31]. CCL22 levels increased only in susceptible LIII animals, indicating that this chemokine should be further studied in animal models and in RA.

CCL6, homolog to human CCL23, is a potent macrophage chemoattractant during skin wound healing [32], is expressed in the bone, attracting osteoclasts [33], and is also involved in angiogenesis [34]. mCCL6/hCCL23 is secreted by mouse and human eosinophils [35, 36], cells that were recruited to the PerC of pristane-injected mice. CCL6 levels increased in both HIII and LIII mice at 7 days but only remained elevated in HIII, after 35 days. Serum CCL23 has been described as a RA activity biomarker [37]; however, murine CCL6 has not been ascribed a role in murine arthritis models.

CCL5 binds CCR1/3/4/5 and is elevated, together with CCL2 and other chemokines, in human RA synovium [38]. This chemokine induces metalloproteinases such as MMP-1 and MMP-13 in RA synovial fibroblasts and thus is involved in cartilage destruction in the late stages of human disease [39]. On the other hand, mice deficient for CCR5, one important CCL5 receptor, showed exacerbated proteoglycan-induced arthritis [40]. CCL5 production 7 days postpristane injection was more intense in the peritoneum of LIII mice, and infiltrating leukocytes also expressed more *Ccr5* mRNA at this timepoint.

In short, several CC chemokines with established roles in clinical human RA also participate in the early preclinical phase of PIA.

Inflammatory and immune cells infiltrating the PerC of LIII mice may express more than one chemokine receptor, usually in a sequential fashion for extravasation into tissues and homing to secondary lymphoid tissues, in a steady state and inflammatory conditions [41]. *Ccr2* and *Ccr3* expression increased in both strains, while *Ccr5* increased in LIII mice only. These analyses are limited in that expression was measured in the whole peritoneal cell infiltrate. Further immunophenotyping of sorted cell populations would address this issue.

Mice selected for maximal acute inflammation, homozygous for *Slc11a1* R (AIRmax^RR^) or S (AIRmax^SS^) alleles, also differ in PIA susceptibility [42]. *Slc11a1* is involved in macrophage activation, and the S allele is associated with upregulation of chemokine and chemokine receptor genes in peritoneal macrophages of AIRmax^SS^ mice during the late clinical phase of PIA [43]. The PerC of pristane-injected LIII mice shared a similar CC chemokine profile to AIRmax^SS^, suggesting that these mediators may be relevant throughout PIA development. On the other hand, levels of the PMN-specific chemokine CXCL1 increased in both HIII and LIII mice in the early preclinical phase, while expression was downregulated in peritoneal macrophages of both AIRmax^SS^ and AIRmax^RR^ mice. This may reflect the early acute phase analyzed in HIII and LIII mice, as opposed to the late, chronic phase of PIA studied in AIRmax^SS^ and AIRmax^RR^. HIII and LIII mice both carry the *Slc11a1* R allele (unpublished results), suggesting that their genetic background contains factors with more profound effects in PIA susceptibility.

In pristane lupus, resident and pristane-elicited peritoneal macrophages from BALB/c mice secrete CCL2, CCL3, CXCL1, and IL-6 when stimulated with TLR7 or TLR4 ligands [44]. In our model, pristane induced similar CCL2 and CXCL1 levels in both HIII and LIII mice. However, CCL3 was only produced by LIII mice, while IL-6 levels were higher in HIII animals, suggesting that the peritoneum of the two strains contains functionally distinct cell populations. Also, joint damage was assessed in this work using BALB/c mice. Pristane induced only mild signs of arthritis after 6 months, in contrast to the severe arthritis of LIII mice observed as early as 45 days [17]. Expression of interferon-induced genes (ISGs) such as *Ccl2* and *Mx1* increases before the onset of pristane lupus symptoms [45]. In our work, mRNA levels of these ISGs increased in both HIII and LIII strains during the preclinical phase, suggesting that type I interferon activity may be an intrinsic effect of pristane. Thus, the importance of the type I interferon signature, which is still under debate for human RA [46], could not be established in the present study for PIA.

Our results showed a significant divergence in mononuclear (macrophage and DC) numbers in the PerC of HIII and LIII mice 7 days postpristane injection. While we did not define the proportion of resident (LPMs) and infiltrating monocytes and macrophages (SPMs), HIII macrophages were depleted 7 days after pristane, similar to C57BL/6 mice [47], a strain not reported as PIA susceptible.

IL-6, a pleiotropic cytokine, is involved in numerous immune and inflammatory processes, as well as in processes that resolve or counteract inflammation, due to dual signalling—classic and trans—mediated by membrane-bound and soluble IL-6R, respectively (reviewed in [48]). Arthritis in TNF-*α* transgenic mice was not affected by IL-6 deficiency, while CIA was abolished [49], possibly involving inhibition of Treg differentiation by this cytokine in the latter model [50]. High PerC IL-6 levels were detected in BALB/c mice, 1-4 months postpristane injection [51]. In agreement with these results, peritoneal IL-6 increased only 35 days postpristane injection, however, with much higher intensity in the PIA-resistant HIII strain.

IL-1 family cytokines are involved in acute inflammation, resistance to microbial pathogens, and among other functions, and act in clinical human RA, which may be treated with Anakinra (recombinant IL-1Ra, reviewed in [52]). Our results showed that IL-1Ra levels were highly elevated in both HIII and LIII mice and the cytokines antagonized by IL-1Ra (IL-1*β* and IL-1*α*) were either unaltered or slightly elevated in HIII mice. On the other hand, IL-18, which is not antagonized by IL-1Ra, increased in susceptible LIII mice, suggesting a role for this cytokine in the early events of PIA development.

IL-12 family members such as IL-12p40, IL-12p70, and IL-23 are produced by activated leukocytes including macrophages, neutrophils, and DCs [53]. The role of IL-12p70 in CIA, depends on the phase of the disease, exacerbating symptoms in the preclinical phase and showing an “anti-inflammatory” effect during established disease [54]. Contrary to this finding, peritoneal IL-12p70 levels were unaltered by pristane in HIII and LIII mice, suggesting a minor role for this cytokine in early PIA development. The relative effect of IL-12p70 and IL-23, which may be considered a proxy for the Th1-IFN-***γ*/**Th17-IL-17 balance, as well as direct actions of IL-23 on osteoclastogenesis and IL-1β/IL-6 production, might determine the fate of the joints [55]. IL-23 was slightly elevated in LIII mice, suggesting that this balance would favor IL-17, IL-1*β*, and IL-6 production. However, neither IL-17 nor IL-1*β* levels increased in the PerC of HIII and LIII mice and IL-6 increased more intensely in the resistant HIII strain. IL-12p40, which is known to be produced in excess over IL-12p70 [53], may act as an IL-12 antagonist but, as the IL-12p80 homodimer, may promote the homing of activated DCs to lymph nodes [56]. This mechanism could facilitate DC-mediated activation of autoimmune T cells in LIII mice.

## 5. Conclusions

In summary, the unique HIII and LIII strains, with extreme divergence in PIA susceptibility, are useful tools for unravelling the fundamental early events that determine the fate of the joints in this model. Despite the complexity of the peritoneal microenvironment and the reaction induced by pristane, our results show that early production of CC chemokines, which shape the peritoneal inflammatory phenotype of LIII mice, may play an important role in the subsequent autoimmune responses leading to PIA susceptibility, and are potential biomarkers. These results also might provide novel insights into the etiology of disease in this model and eventually in human disease.

## Figures and Tables

**Table 1:** Primer pairs used in qRT-PCR assays.

Gene	Forward primer (5′-3′)	Reverse primer (5′-3′)
*Ppia*	AGCGTTTTGGGTCCAGGAAT	AAATGCCCGCAAGTCAAAAG
*B2m*	CCCCACTGAGACTGATACATACG	CGATCCCAGTAGACGGTCTTG
*Rps29*	TCTACTGGAGTCACCCACGGAAGT	GTCAGTCGAATCCATTCAAGGTCGC
*Gapdh*	AGACGGCCGCATCTTCTTGTGC	TACGGCCAAATCCGTTCACACCG
*Tbp*	GACCAGAACAACAGCCTTCCACCT	TGTGGAGTAAGTCCTGTGCCGTAAG
*Ccr1*	ACTCTGGAAACACAGACTCACTG	GTTGTGGGGTAGGCTTCTGT
*Ccr2*	TTGACCACCTTCCAGGAATC	CTGCATGGCCTGGTCTAAGT
*Ccr3*	TGTTATCTCTGTTTCATTAGCAGTG	CATAGGGTGTGGTCTCAAAGC
*Ccr4*	AGAAGAGCAAGGCAGCTCAA	GGTGGTGTCTGTGACCT
*Ccr5*	CCAGAGGAGGTGAGACATC	GCAGGGTGCTGACATACCA
*Cxcr2*	AGCCACTCTGCTCACAAACA	CCACCTTGAATTCTCCCATC
*Ccl2*	GCCTGCTGTTCACAGTTGC	TCATTGGGATCATCTTGCTG
*Mx1*	GACCATAGGGGTCTTGACCAA	AGACTTGCTCTTTCTGAAAAGCC

## Data Availability

The data files used to support the findings of this study are available from the corresponding author upon request.

## Supplementary Materials

Supplementary 1 Figure S1: peritoneal CXCL1 levels, *Cxcr2*, *Ccl2*, and *Mx1* gene expression in pristane-injected HIII and LIII mice.

Supplementary 2 Figure S2: peritoneal cytokine levels in pristane-injected HIII and LIII mice.
