# Supplementary material for: Early Peritoneal CC Chemokine Production Correlates with Divergent Inflammatory Phenotypes and Susceptibility to Experimental Arthritis in Mice
Source: J Immunol Res. 2019 Feb 26;2019:2641098. doi: 10.1155/2019/2641098 (PMC6413398; doi:10.1155/2019/2641098)
Supplement: Supplementary 1 — Figure S1: peritoneal CXCL1 levels, Cxcr2, Ccl2, and Mx1 gene expression in pristane-injected HIII and LIII mice. [file 2641098.f1.pdf]

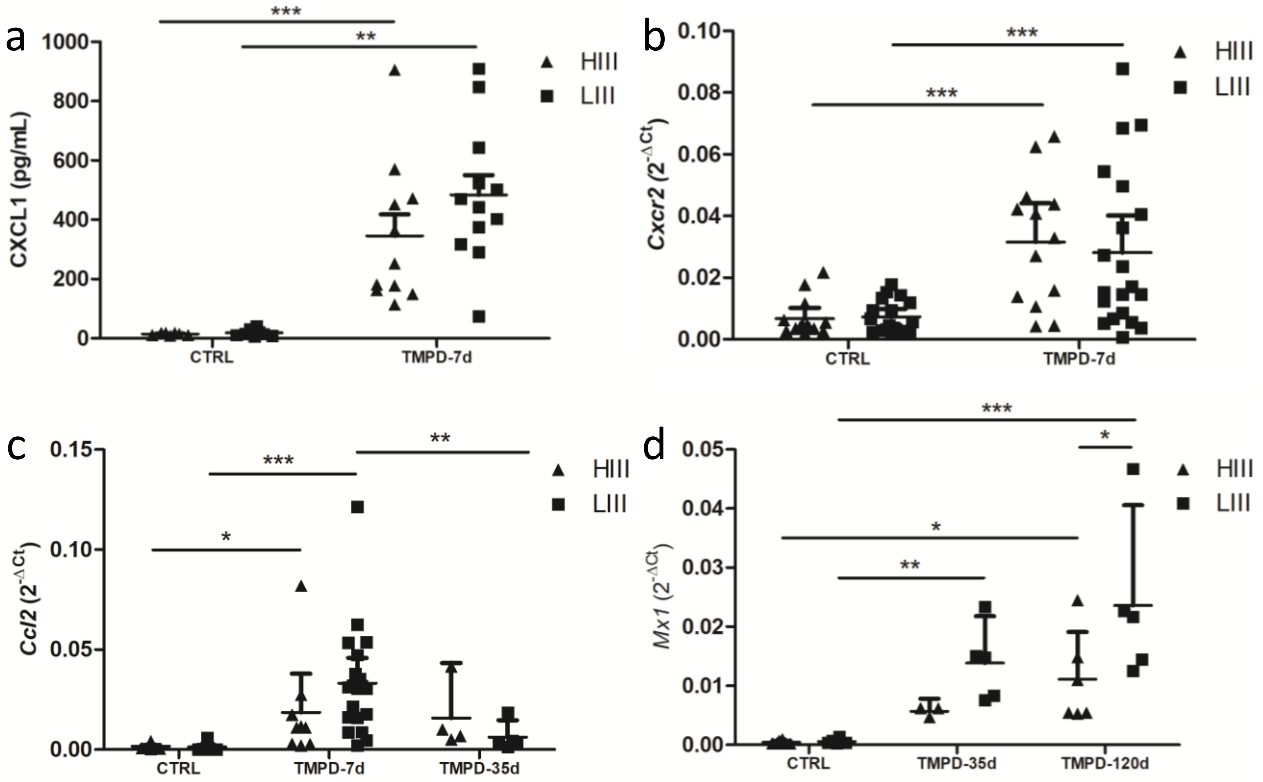

Figure S1: PerC CXCL1 levels (a), *Cxcr2* (b), *Ccl2* (c) and *Mx1* (d) gene expression in peritoneal infiltrating leukocytes of HIII and LIII mice injected with pristane. Mice were injected with pristane (TMPD) or saline (CTRL) and peritoneal lavage fluid was harvested after 7 (a-b), 7 and 35 (c) or 35 and 120 days. Bars represent mean±95% confidence interval of 1-3 experiments with 4-6 animals/group (two-way ANOVA followed by Bonferroni post-tests). *p<0,05; **p< 0,01; ***p<0,001.
