# Supplementary material for: Early Peritoneal CC Chemokine Production Correlates with Divergent Inflammatory Phenotypes and Susceptibility to Experimental Arthritis in Mice
Source: J Immunol Res. 2019 Feb 26;2019:2641098. doi: 10.1155/2019/2641098 (PMC6413398; doi:10.1155/2019/2641098)
Supplement: Supplementary 2 — Figure S2: peritoneal cytokine levels in pristane-injected HIII and LIII mice. [file 2641098.f2.pdf]

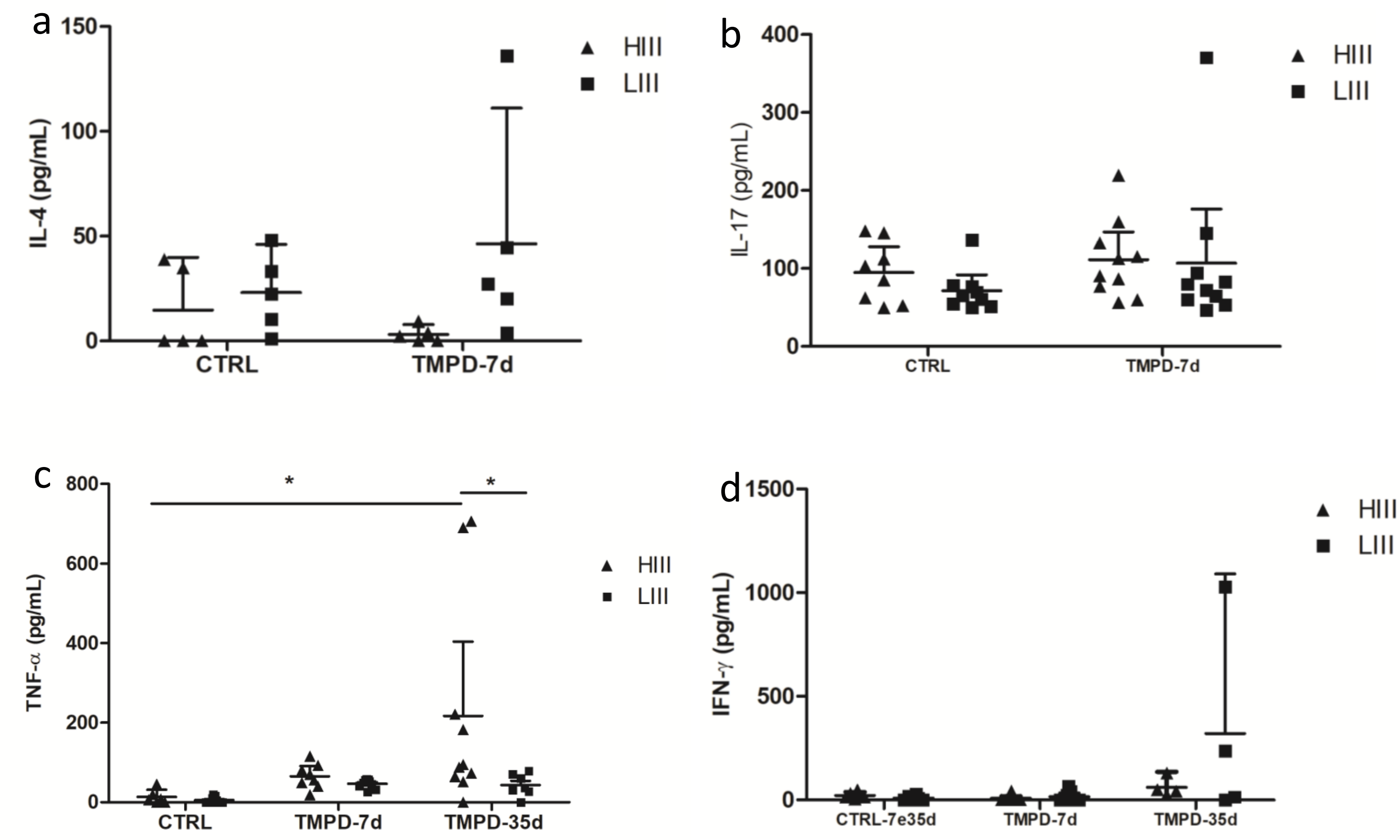

Figure S2: PerC cytokine levels of HIII and LIII mice injected with pristane. Mice were injected with pristane (TMPD) or saline (CTRL) and peritoneal lavage fluid was harvested after either 7 days (IL-4 - a; IL-17 - b) or 7 and 35 days (TNF-α - c; IFN-γ - d). Bars represent mean±95% confidence interval of 1 or 2 experiments with 4-6 animals/group (two-way ANOVA followed by Bonferroni post-tests). *p<0,05; **p< 0,01; ***p<0,001.
